# Mobile videoconferencing for enhanced emergency medical communication - a shot in the dark or a walk in the park? ‒‒ A simulation study

**DOI:** 10.1186/1757-7241-22-35

**Published:** 2014-06-02

**Authors:** Sigurd Melbye, Martin Hotvedt, Stein Roald Bolle

**Affiliations:** 1 Faculty of Health Sciences, UiT The Arctic University of Norway, N-9037 Tromsø, Norway; 2 University Hospital of North Norway, Norwegian Centre for Integrated Care and Telemedicine, P.O. Box 35, N-9038 Tromsø, Norway

**Keywords:** Emergency medicine, Emergency telehealth, Telemedicine, Videoconference, E-health, M-health, Videoconferencing, Emergency medical services, Cellular phone, Remote consultation

## Abstract

**Background:**

Videoconferencing on mobile phones may enhance communication, but knowledge on its quality in various situations is needed before it can be used in medical emergencies. Mobile phones automatically activate loudspeaker functionality during videoconferencing, making calls particularly vulnerable to background noise. The aim of this study was to investigate if videoconferencing can be used between lay bystanders and Emergency Medical Dispatch (EMD) operators for initial emergency calls during medical emergencies, under suboptimal sound and light conditions.

**Methods:**

Videoconferencing was tested between 90 volunteers and an emergency medical dispatcher in a standardized scenario of a medical emergency. Three different environments were used for the trials: indoors with moderate background noise, outdoors with daylight and much background noise, and outdoors during nighttime with little background noise. Thirty participants were recruited for each of the three locations. After informed consent, each participant was asked to use a video mobile phone to communicate with an EMD operator. During the video call the EMD operator gave instructions for tasks to be performed by the participant. The video quality from the caller to the EMD was evaluated by the EMD operator and rated on a five step scale ranging from "not able to see" to "good video quality". Sound quality between participants and EMD operators was assessed by a method developed for this trial. Kruskal – Wallis and Chi-square tests were used for statistical analysis.

**Results:**

Video quality was significantly different between the groups (p <0.001), and the nighttime group had lower video quality. For most sessions in the nighttime group it was still possible to see actions done at the simulated emergency site. All participants were able to perform their tasks according to the instructions given by dispatchers, although with a need for more repetitions during sessions with much background noise. No calls were rated by dispatchers as incomprehensible due to low sound quality and only 3% of the calls were considered *somewhat difficult* or *very difficult to understand*.

**Conclusions:**

Videoconferencing on mobile phones can be used for the initial emergency call during medical emergencies also in suboptimal conditions.

## Background

Accidents, cardiac arrest and other medical emergencies are major contributors to death today [1,2]. Lay bystanders can in these situations help to initiate early treatment, which in turn may prevent death [3]. Measures to improve emergency medical treatment in the early phase may therefore help to save lives and reduce suffering. In Norway, the Emergency Medical Dispatch (EMD) Centres assist bystanders in medical emergencies via telephone [4]. Today’s communication systems between lay bystanders and the EMD Centres are based on audio-only telephone calls [5] and are not adapted to the possibilities offered by smart phones – such as videoconferencing.

Several studies have examined the use of videoconferencing in medical emergencies. Video communication may improve the operators understanding of the callers situation, and thereby improve the instructions they give [3,6]. In simulated trials, video images during instructions for cardiopulmonary resuscitation (CPR) from EMD operators to lay bystanders have improved the quality of mouth-to-mouth ventilation, increased the proportion of opened airways and increased ventilation volume [7]. EMD operator assisted CPR through video communication improved the callers’ self-reported confidence [6]. Such help could possibly increase the number of lay bystanders who start CPR before the ambulance arrives.

Previous studies on the use of video mobile phones during medical emergencies have been conducted under good light conditions and in quiet environments. Many calls to EMD centres come from poorly lit places, with varying degrees of background noise. When mobile phones are used for videoconferencing, loudspeaker functionality is automatically activated, which makes calls particularly vulnerable to background noise. Insufficient bandwidth may cause interruptions in the video flow, thus decreasing the usefulness of the image. A potential new video-based EMD service must take these aspects into account. We wanted to investigate if videoconferencing can be used between lay bystanders and EMD centres during medical emergencies under suboptimal sound and light conditions.

## Methods

### Setting

Sound and video quality was tested during 90 sessions of a simulated medical emergency. In each session, a volunteer communicated through a video mobile phone with an EMD operator. We chose three different locations for the experiments in public areas in Tromsø, Norway, in order to assess whether different environments around the patient affect the perception of sound and video quality. Thirty calls were completed in each location; first 30 calls indoors in a shopping centre with good lighting and moderate background noise, then 30 calls outdoors next to a busy road during daylight and with much background noise, and finally 30 calls outdoors at night with poor lighting and minimal background noise. In total, 90 volunteers participated in one session each.

Volunteers were recruited from a stand on each location and given oral and written information about the project. Persons not speaking Norwegian or who had seen others participate in the study were not included. After verbal acceptance to participate in the study, the volunteer filled out a form with information about age, gender, highest completed education, and whether they previously had called an EMD centre.

Two fifth year medical students (SM and MH), played the role as either the EMD operator or the assistant on the medical emergency site. The latter recruited volunteers on the simulated emergency site, assisted the volunteer to set up a video call to the EMD centre through the mobile phone and made notes of comments from the volunteers. For each location the roles of the students were switched after 15 sessions.

### Scenario

**Figure 1:**
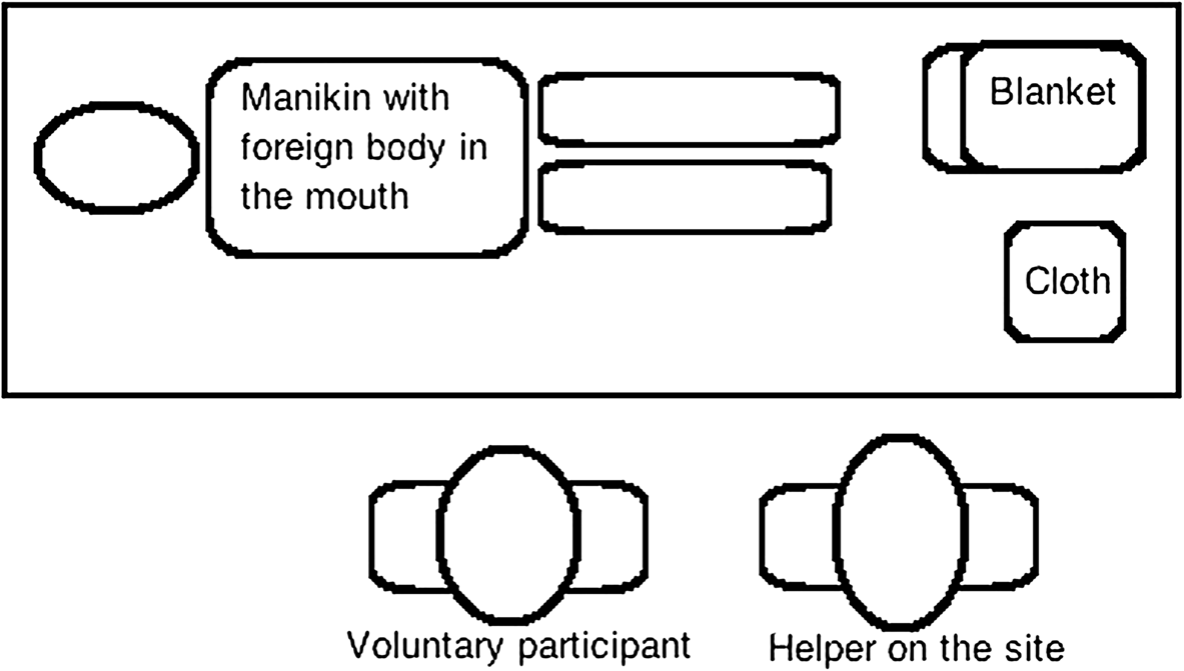
Schematic view of the simulated emergency site.

At the simulated medical emergency site a manikin (Laerdal Resusci Anne Manikin, Laerdal, Stavanger, Norway) was placed on a table. A blanket and a cloth were placed next to the manikin, and a foreign body (coin) was placed in the manikin’s mouth (Figure 1).

Prior to the trial a sheet of paper containing patient information was generated for each session to be read by the volunteer. The patient information contained first name, surname, age and street address. The data used for generation of names was randomly chosen from Statistics Norway’s lists of female names and surnames used in Norway by 200 or more individuals [8]. Patient age was chosen by random by computer software (18–75 years) and addresses were randomly chosen from a list of street addresses in the municipality of Tromsø. The items were randomized, joined to a sentence and printed using spreadsheet software. No address, first name or last name was reused. Thus the EMD operator was not able to predict the patient information before it was read by the volunteer.

**Figure 2:**
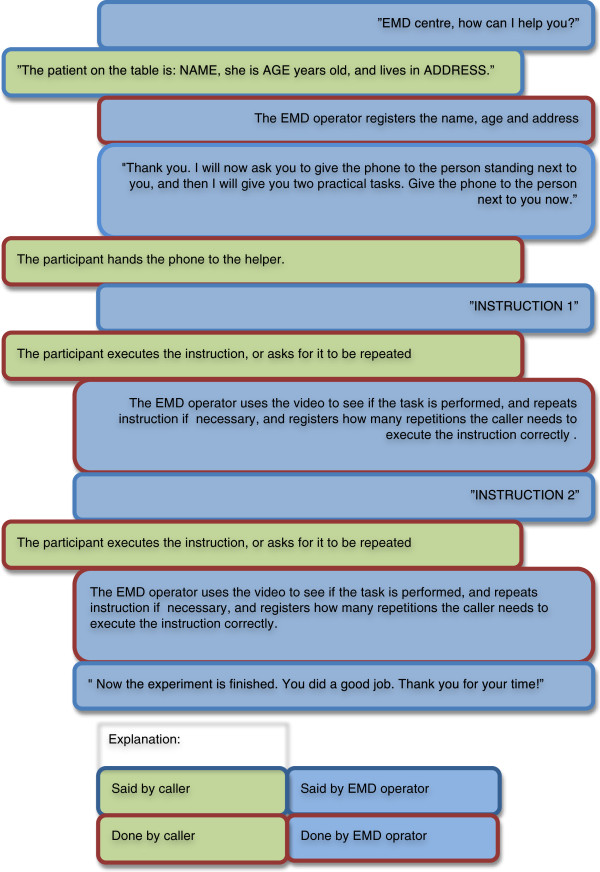
Flowchart of the communication between the volunteer and the EMD operator when using mobile phone videoconferencing in a simulated medical emergency.

The Norwegian Index of Medical Emergency Assistance contains instructions to be given by EMD operators to callers [9]. Ten such instructions were selected for this trial (Appendix 1), and for each session two instructions were randomly chosen to be given to the caller during the videoconference.After recruitment, the volunteer was given the paper sheet with patient information, and the following oral instruction: "We are going to simulate a video call with the Emergency Medical Dispatch Centre (113). You should read the text on this sheet of paper to the person in the other end. Then follow the instructions given by the person you are talking to on the phone. During this test you should only speak with the person at the Dispatch Centre." The video enabled mobile phone was connected to the EMD operator and handed to the volunteer, and communication started (Figure 2).

### Communication technology

On the simulated emergency site we used a smartphone for communication with the EMD operator, the Apple iPhone 5 ™ (Apple Inc®, Cupertino, CA, USA), with a 1.3 Mega pixel front camera. We chose to only use the front camera as it is started automatically when the user initiates a videoconference, and it also allows the participants to see each other. The front camera has no flash and the transmitted image is therefore dependent on light from the surroundings. Videoconferencing on mobile phones can be used on either the 3G or the 4G mobile broadband. The 3G network provides a maximal upload/download speed of 10/10 Mbit/s, while the 4G network provides a maximal upload/download speed of 10/40 Mbit/s. We chose to disable the 4G transceiver as this network had limited coverage at the time of study.

We simulated an EMD centre using an Apple MacBook Pro™ (Apple Inc®, Cupertino, CA, USA) with a 1.3 Megapixel camera and a WiFi network with 10 Mb/s wireless upload and download speed. The EMD centre was not co-located with the simulated emergency site. In order to conduct video communication between phone and computer, we used the Apple FaceTime TM software (Apple Inc®, Cupertino, CA, USA), in which both audio and video signals are transferred over the internet.

### Data collection

The EMD operator wrote down the information read by the volunteer (patient name, age and address). In order to use the portion of correctly recorded information as a measurement of sound quality, the EMD operator was not allowed to ask for repetitions of the patient information. The sound quality was also scored by the EMD operator on a five step scale ranging from "incomprehensible" to "very easy to understand". The EMD operator gave the volunteer two instructions, and the number of repetitions for each instruction to be performed correctly was recorded.

The quality of the video from the caller to the EMD was evaluated by the EMD operator and rated on a five step scale ranging from "not able to see" to "good video quality". Additionally the EMD operator recorded any interruption in the video flow. The quality of the video from the EMD operator to the caller was not assessed.

### Data analysis

The notes from the EMD operator was compared to the patient information sheet from the simulated emergency site, with relation to patient name, age and address, and the registrations of the EMD operators rated as either right or wrong. Descriptive statistics were used to summarize data, and Kruskal – Wallis and Chi-square tests used for statistical analysis with an alpha level of 0.05. For calculation of chi-square, groups were joined due to small data sets. For statistical calculations, we used the software packages SPSS (IBM Corporation, Armonk, NY, USA) and Excel (Microsoft Corporation, Redmond, Washington, USA).

### Ethics

The volunteers received oral and written information about the study and how to contact the research group for questions or results, after which they gave an oral consent to participate in the study. Neither sound nor image from the conversations were recorded or stored. The forms that were filled out contained no information that could be linked to the volunteers and forms were destroyed at the end of the data collection. The regional ethics committee confirmed that ethical approval was not required for this project.

## Results

The median age of the volunteers were 31 years, 37% were females, 41% had previously called an EMD centre, and 63% had higher education (Table 1(a)). There were no significant differences regarding these values between the *indoors*, *daylight and nighttime* groups. The groups differed regarding the level of education (p = 0.042), as the nighttime group had a higher proportion of participants with college or university degree.

**Table 1:** Data from 90 calls using mobile phone videoconferencing for communication between volunteers and an emergency medical dispatch centre in a simulated medical emergency

	**Indoors (n = 30)**	**Daylight (n = 30)**	**Nighttime (n = 30)**	**Total (n = 90)**	**p value**^**1**^
**a: Demographics of the 90 volunteered participants.**					
Age, median (min, max)	32.5 (14, 78)	34.0 (15, 75)	28.0 (21, 66)	31.0 (14, 78)	0.839^2^
Female volunteers (n (%))	8 (27)	13 (43)	12 (40)	33 (37)	0.366
Volunteers who has called an EMD center previously (n (%))	12 (40)	13 (43)	12 (40)	37 (41)	0.955
Volunteers with college or university degree (n (%))	18 (60)	18 (60)	21 (70)	57 (63)	0.042*
**b: Number of elements correctly registered by EMD operator. When calculating p value the top three alternatives where combined.**					
No elements (n (%))	0 (0)	0 (0)	0 (0)	0 (0)	0.065
One element (n (%))	5 (17)	0 (0)	3 (10)	8 (9)	
Two elements (n (%))	11 (37)	8 (27)	6 (20)	25 (28)	
Three elements (n (%))	14 (46)	22 (73)	21 (70)	57 (63)	
**c: Sound quality as scored by EMD operator. When calculating p value the top four alternatives where combined.**					
Incomprehensible (n (%))	0 (0)	0 (0)	0 (0)	0 (0)	0.153
Very difficult to understand (n (%))	0 (0)	1 (3)	0 (0)	1 (1)	
Somewhat difficult to understand (n (%))	0 (0)	0 (0)	2 (7)	2 (2)	
Fairly easy to understand (n (%))	3 (10)	5 (17)	7 (23)	15 (17)	
Very easy to understand (n (%))	27 (90)	24 (80)	21 (70)	72 (80)	
**d: Video quality as scored by EMD operator. When calculating p value the top two alternatives where combined.**					
Not able to see instructions executed (n (%))	0 (0)	1 (3)	1 (3)	2 (2)	<0.001*
Low (n (%))	0 (0)	1 (3)	8 (27)	9 (10)	
Pretty low (n (%))	0 (0)	3 (10)	13 (43)	16 (18)	
Pretty good (n (%))	11 (37)	7 (23)	8 (27)	26 (29)	
Good (n (%))	19 (63)	18 (60)	0 (0)	37 (41)	

Three different locations were used as the emergency site in order to study the influence of light and noise on the video call: *indoors* with good light conditions and moderate background noise, outdoors in *daylight* with background traffic noise, and outdoors at *nighttime* with poor light conditions and very little background noise.

^1^Chi-square test was used for calculating p value, except regarding Age.

^2^For Age, Kruskal-Wallis test was used for calculating p value.

*Significant on a 5% level.

The volunteers executed the instructions from the EMD operator without need for repetitions in 80% of the calls, 77% in the indoors group, 67% in the daylight group, and 97% in the nighttime group, with statistical difference between groups (p = 0.013). For all calls both instructions were completed correctly after a maximum of one repetition.

In 63% of the calls, all patient information was perceived correctly by the EMD operator (Table 1(b)). Only 3% of the calls were considered *somewhat difficult* or *very difficult to understand*. No calls were rated as incomprehensible due to low sound quality (Table 1(c)). Sound quality from volunteers to the EMD centre was not different between the three groups.

In the assessment of video quality there was a significant difference in quality between the groups (p <0.001, Table 1(d)). The video quality was lower in the nighttime group, but in only two of the nigthtime sessions were the EMD operators "not able to see instructions executed". For all other sessions it was possible to see on screen what took place at the rescue scene.

There were interruptions of the video flow, such as image freeze, in 21% of calls, but the frequency of interruptions did not differ significantly between groups (p = 0.282). In most sessions the interruptions lasted less than one second, but in one session the number and length of interruptions made it difficult to see what was done at the simulated emergency site.

During the experiments we received several comments from volunteers. Some commented on the reassuring aspect of visual communication: *"I would feel reassured if the EMD operator could see whether I did the right things - and possibly correct me"* (man, age 60), and "*It felt right to see the operator in a situation like this*" (woman, age 23). One person commented on the extra time needed for the call to connect: "*I would not like to wait for the phone to connect for the video communication in a stressful and critical situation*" (woman, age 45).

We also received comments on the quality of the technology: "*The quality of the video and sound was surprisingly good. This should be available soon*" (man, age 40).

## Discussion

This study compared the quality of videoconferencing for volunteers communicating with an EMD operator in simulated medical emergencies at three different locations: indoors, outdoors with daylight and outdoors at nighttime. The EMD operator was able to see what was done in almost all scenarios, although with greater difficulty during poor light conditions. In spite of the great differences in background noise between the three groups, there was no significant difference in the EMD operator’s perception of sound quality. The volunteers were in all cases able to hear instructions from the EMD operator, although instructions were more often repeated for calls with much background noise.

A measure of sound quality from the caller to the EMD was the number of information items (name, age, address) registered correctly by the EMD operator without asking for repetition of information. A low success rate can be interpreted as poor sound quality or disturbance (e.g. background noise). In today’s EMD service, sound is the only information channel between a caller and the EMD operator, which at times is challenging [5]. We found that in nearly 40% of the calls, one or more information elements were not registered correctly, but in a real situation the EMD operator would ask for confirmation that the information is correctly perceived, or ask for repetition. In our study, 97% of calls was perceived by the EMD operator as "very easy" or "fairly easy to understand", and therefore the errors done when collecting patient information may be due to the caller reading out information quickly or indistinct, as the EMD operator in this study was not allowed to ask for repetitions.

The sound quality from the EMD operator to the caller was measured as the need to repeat instructions to the caller. The need for repetitions was lowest in the nighttime group, which may be explained by less background noise in these sessions. Loudspeaker functionality has several advantages, as the caller can put the phone down and still hear what is being said, and the EMD operator can communicate with several persons at the same time. The latter has been perceived beneficial in other studies [3]. Even with much background noise, the need for repetitions was low, and loudspeaker functionality is therefore a feature that may be well suited for use in emergency medical situations.

The video quality from the caller to the EMD was measured as the EMD operator’s subjective perception. There was a significant difference between the three groups, and the *nighttime* group, where light conditions were poor, had the lowest quality. If camera light had been activated, results might have been better for the nighttime group. However, even a low quality-image has been described as valuable for the EMD operator in a previous study [6]. In our study, only 1 out of 30 calls in the *nighttime* group was rated as "not possible to see the execution of instructions".

In this study, we did not measure any time intervals - e.g. the duration of the sessions, the time until the first action was performed and the time used to connect the call. Previous studies have demonstrated some improvements in time factors and resuscitation quality when video conferencing has been used for simulated emergencies [3,7,10,11], but these aspects should be furthered studied when mobile video conferencing is introduced for real emergency calls. Data privacy and information security for video calls must be handled for video calls. A previous risk assessment concluded that no risks to information security were found that would advocate against the use of video calls between lay bystanders and EMD Centres, given proper implementation [12].

During videoconferencing, low capacity of the network can lead to a partial loss of the video information, which is perceived as interference in the video image. This may lead to loss of important information and has earlier been a problem with videoconferencing on mobile networks with low bandwidth. In this study the EMD operator registered all cases of video disturbance, also those of short duration, and we found absence of this type of interference in almost 80% of the video calls. The EMD operator’s understanding of the situation was affected by video interference in only one of the ninety sessions.

The demographics for the three groups were similar except for the level of education, which was higher in the nighttime group, which may partly explain why this group needed fewer repetitions of instructions. In this simulation study we did not want poor knowledge about the telephone to influence the results, therefore the assistant held the telephone used by the volunteer during most of the scenario. In a real setting less favourable results may therefore be expected. We did not standardize the distance between the mobile phone and the volunteer, which may have influenced the perception of both sound and video quality. The light conditions changed with the weather during the nighttime calls, which may have influenced the perception of video quality. If we had allowed for the use of the camera flashlight it may have given better image details and better results in poor light conditions. The order of the sessions in this trial was not randomized. The EMD operator may therefore have performed better during the last sessions due to learning. The roles as EMD operators were played by medical students without experience from real life EMD centres. Experienced EMD operators may have rated the quality of information from the volunteers differently. In spite of these study limitations, our study suggests that today’s mobile phones and networks transfers sufficient quality of video and sound for use during medical emergencies. Other communication technologies, such as other smartphones or networks and different communication software may give different results.

## Conclusion

Videoconferencing between lay bystanders and EMD operators can contribute to a richer communication than audio-only calls. Mobile phone videoconferencing activates loudspeaker functionality and makes the call vulnerable to background noise. This study has shown that it is possible to receive video during nighttime in poor light conditions, and that loudspeaker functionality can be used in noisy environments. We conclude that videoconferencing on mobile phones can be used between lay bystanders and an EMD operator during pre-hospital medical emergencies, also in suboptimal sound and light conditions.

## Appendix 1

The instructions that were given to the volunteer were taken verbatim from the Norwegian Index of Medical Emergencies [9] and randomized from this selection of 10:

1. Undress the patient immediately and as much as possible.

2. Wrap a blanket around the patient.

3. Place something soft under the head of the patient.

4. Pull away from the patient.

5. Keep the head of the patient stable in relation to body.

6. Pinch the nostrils of the patient.

7. Try to remove the foreign object from the mouth of the patient.

8. Loosen the patient’s tight clothing.

9. Wipe the nose and mouth of the patient with a cloth.

10. Tilt the head of the patient gently backwards.

## Abbreviations

EMD: Emergency Medical Dispatch; CPR: Cardio-Pulmonary Resuscitation.

## Competing interest

The authors declare no conflicts of interest.

## Author’s contribution

SM and MH have contributed to the conception and design of the study, acquisition and analysis of data and have drafted the manuscript. SRB has contributed to the conception and design of the study, has helped to analyze the data and has critically revised the manuscript for important intellectual content. All authors read and approved the final manuscript.

## Author’s information

SM and MH are both sixth year medical students at the University of Tromsø, Tromsø, Norway and cofounders of Forbedring Tromsø, a patient safety and quality improvement student organization.

SRB (MD, PhD) is a specialist in anaesthesiology at the University Hospital of North Norway, and a researcher in the Specialist Health Services at the Norwegian Centre for Integrated Care and Telemedicine, Tromsø, Norway. His research has contributed to the development of audio and video communication for medical emergencies.
